# Oat β-Glucan Attenuates Diabetic Liver Injury by Improving the Gut Microbiota

**DOI:** 10.3390/nu18142313

**Published:** 2026-07-14

**Authors:** Xin Wu, Yanfei Jiang, Can Liu, Deying Zhu, Ruixue Mao, Lixia He, Ruisheng Fu, Yong Li, Meihong Xu

**Affiliations:** 1 Department of Nutrition and Food Hygiene, School of Public Health, Peking University, Beijing 100191, China; 2 Department of Biochemistry and Molecular Biology, Beijing Key Laboratory of Protein Posttranslational Modifications and Cell Function, School of Basic Medical Science, Peking University Research Center on Aging, Peking University, Beijing 100191, China; 3 Division of Molecular and Cellular Oncology, Dana-Farber Cancer Institute, Brigham and Women’s Hospital, Harvard Medical School, Boston, MA 02115, USA

**Keywords:** oat β-glucan, diabetic liver injury, gut microbiota, inflammatory response

## Abstract

Objectives: This study investigated the effects of oat β-glucan on liver injury and the gut microbiota in a rat model of a high-fat diet combined with streptozotocin (STZ)-induced type 2 diabetes. Methods: To achieve this, specific-pathogen-free (SPF) male Sprague-Dawley (SD) rats were utilized and initially acclimated to the laboratory environment for one week. Following the stabilization period, 12 rats were randomly selected as the normal control (CON) group and fed a standard chow diet, while the remaining rats were fed a high-fat diet for 4 weeks and then were induced via intraperitoneal (i.p.) injection of multiple low doses of STZ (30 mg/kg) in freshly prepared sodium citrate–citric acid buffer (pH 4.5) once a week for two consecutive weeks. Blood glucose levels were measured using a glucometer, and rats with fasting blood glucose levels exceeding 11.1 mmol/L for 7 consecutive days were confirmed as diabetic. Subsequently, 48 confirmed diabetic rats were then randomly divided into four subgroups (*n* = 12 each): the diabetic model (DM) group, Oat β-Glucan Low-Dose (OGL) group, Oat β-Glucan Middle-Dose (OGM) group, and Oat β-Glucan High-Dose (OGH) group. The rats in the OGL, OGM, and OGH groups were gavaged with oat β-glucan at doses of 0.275 g/kg BW, 0.55 g/kg BW, and 1.10 g/kg BW, respectively, daily for 10 weeks, while the CON and DM groups received an equivalent volume of distilled water. Results: Following the 10-week intervention, the rats were sacrificed for multi-indicator assessments. Oat β-glucan treatment significantly improved hyperglycemia and alleviated hepatic injury. Histological analysis confirmed that oat β-glucan alleviated pathological liver damage. Furthermore, oat β-glucan effectively reshaped the gut microbiota by reducing the abundance of *Enterococcaceae* and increasing the abundance of beneficial taxa, including *Ruminococcaceae*, *Lachnospiraceae*, and *Christensenellaceae*. Compared with the DM group, the OGH group exhibited a significant reduction in metabolic endotoxemia (serum LPS). Meanwhile, the observed microbial shifts were closely correlated with the downregulation of pro-inflammatory cytokines (TNF-α and IL-6). Conclusions: In conclusion, our findings demonstrate that oat β-glucan exerts a protective effect against diabetic liver injury by modulating gut microbiota composition and suppressing inflammation via the gut–liver axis, highlighting its potential as a functional dietary strategy for diabetes management.

## 1. Introduction

Type 2 Diabetes mellitus (T2DM) is a metabolic disorder characterized by hyperglycemia resulting from impaired insulin-signaling. The International Diabetes Federation (IDF) estimated the global population with diabetes mellitus to be 537 million in 2021, 643 million in 2030 and 783 million in 2045. In addition, approximately 541 million people had impaired glucose tolerance in 2021, and over 6.7 million people aged 20–79 died from diabetes-related causes in 2021 [1]. In China, a combination of population growth [2], aging [3], rising obesity rates [4] and sedentary behaviors [5] led to a substantial increase in the diabetes population from 98.4 million to 140.9 million during 2013–2021 [1]. As one of the diabetic complications, diabetic liver injury is often underdiagnosed due to diagnostic challenges. Diabetic liver injury refers to a common chronic complication secondary to diabetic liver function injury, which is mainly manifested by liver tissue and function lesions, among which metabolic dysfunction-associated steatotic liver disease (MASLD, formerly named NAFLD) is the most common. In 2019, Younossi ZM et al. reported that the estimated global prevalence of MASLD in individuals with T2DM was 55.48% [6]. T2DM drives the progression of MASLD, developing hepatic and extrahepatic adverse outcomes at an accelerated pace [7]. A link between diabetes and liver disease has been suggested in many studies. It is reported that elevated blood sugar could cause liver injury. Studies have shown that patients with type 2 diabetes mellitus (T2DM) face a significantly increased risk of developing MASLD, progressive liver fibrosis, metabolic dysfunction-associated steatohepatitis MASH, and hepatocellular carcinoma [8,9,10]. Liver disease represents a major cause of mortality among individuals with type 2 diabetes mellitus (T2DM). Evidence from multiple population-based observational studies indicates that, compared with non-diabetic individuals, patients with T2DM experience a markedly increased risk of liver-related mortality, with hepatocellular carcinoma (HCC) and metabolic dysfunction-associated steatotic liver disease (MASLD) being the leading causes. For example, a 10-year cohort study in the Piedmont region of Italy, encompassing over four million residents, reported that T2DM patients had a significantly higher risk of liver-related death relative to non-diabetic controls [11]. Although liver damage is highly prevalent among individuals with diabetes, effective pharmaceutical treatments for MASLD and other diabetes-related liver injuries are still lacking [12]. Hence, there is an urgent need to understand the pathogenesis of diabetic liver injury and develop a safe and effective nutritional intervention strategy.

Oats are among the most nutritious whole grains and are the fifth most consumed crop globally. These nutritional benefits are attributed to their diverse composition, including dietary fiber, essential fatty acids, vitamins, functional proteins, and a high content of phenolic compounds [13]. In human nutrition, whole oats are rarely consumed directly; instead, they are widely included in the diet through various processed food products, among which oat bran and rolled oats (oat flakes) possess the highest concentrations of oat components [14]. The whole oat flour normally contains around 12.2% to 16.8% total dietary fiber (TDF). This fiber matrix is distinctly characterized by its composition of insoluble dietary fiber (IDF) and soluble dietary fiber (SDF) fractions. IDF, primarily consisting of cellulose, hemicellulose, and lignin, constitutes the larger fraction at approximately 7.11% to 9.51%. Conversely, SDF accounts for 4.75% to 6.51% [15]. The oat β-glucan content generally ranges from 3% to 8% in whole oats but is significantly concentrated within the internal aleurone and subaleurone cell walls, resulting in a substantially higher density of 7% to 10% in commercial oat bran products [14]. Oat β-glucan, a soluble dietary fiber which is found in the endosperm cell walls of oats, has been reported to exhibit a variety of biological functions, including lowering blood sugar, promoting weight loss, regulating gut microbiota, and preventing cardiovascular disease and cancer [16,17,18,19]. Some studies have shown that oat β-glucan can selectively promote the growth of *Lactobacillus* and *Bifidobacterium* while inhibiting the proliferation of coliforms [20]. The gut microbiota is a complex ecosystem in which microbes interact with each other and with the host [21]. Studies have shown that imbalance in the gut microbiota is closely related to the occurrence of diabetes [22,23]. Previous studies have found that gut microbiota imbalance is associated with the incidence of MASLD, alcoholic liver disease, and primary sclerosing cholangitis [24,25,26], which suggests that oat β-glucan might possess immeasurable potential in attenuating diabetic liver injury by improving the gut microbiota. However, to date, no studies have investigated the protective effect of β-glucan on diabetic liver injury by regulating the gut microbiota.

The aim of the present study was to investigate the potential protective effect of oat β-glucan on diabetic liver injury via the modulation of the gut microbiota. Our findings elucidate the underlying mechanisms and provide a solid foundation for the therapeutic potential of oat β-glucan as a dietary intervention strategy against diabetic liver injury.

## 2. Materials and Methods

### 2.1. Oat β-Glucan

Oat β-glucan was provided by the Inner Mongolia Sanzhuliang Natural Oats Industry Corporation (IMSNOIC) (Hohhot, Inner Mongolia, China) with a purity of ≥95%.

### 2.2. Animals

Healthy SPF male SD rats (200–250 g, 6 weeks old) were obtained from the Peking University Health Science Center for this study. The rats were maintained under controlled environmental conditions (temperature: 22 ± 2 °C; relative humidity: 50–60%) with a 12 h light/dark cycle. All experimental procedures were approved by the Institutional Animal Care and Use Committee of Peking University, and all animals were treated in accordance with the principles of laboratory animal care and the guidelines of the Peking University Animal Research Committee (LA201486).

### 2.3. Experimental Design

After a period of adaptive feeding, 12 rats were randomly assigned to the normal control group (CON, *n* = 12) and fed a standard chow diet. The other rats were fed a high-fat diet (HFD) for 4 weeks. To induce type 2 diabetes mellitus (T2DM), the HFD-fed rats received an intraperitoneal (i.p.) injection of streptozotocin (STZ, 30 mg/kg BW, dissolved in 0.1 M, pH 4.5 sodium citrate–citric acid buffer) once weekly for two consecutive weeks. Concurrently, the CON group was injected with an equivalent volume of sterile saline on the same schedule, serving as a non-diabetic baseline reference. Forty-eight rats were confirmed to have successfully developed type 2 diabetes.

Blood samples were collected from the tail vein, and glucose levels were measured using a glucometer (Johnson & Johnson Medical Ltd., Shanghai, China). Rats exceeding a fasting blood glucose level of 11.1 mmol/L for 7 consecutive days were confirmed as diabetic. Following successful model establishment, the HFD was discontinued, and all diabetic rats were transitioned to the standard chow diet. The 48 diabetic rats were further randomly divided into four subgroups (*n* = 12 per group): the diabetic model (DM) group, the Oat β-Glucan Low-Dose (OGL) group, the Oat β-Glucan Medium-Dose (OGM) group, and the Oat β-Glucan High-Dose (OGH) group. Rats in these groups were administered oat β-glucan via oral gavage at doses of 0.275, 0.55, and 1.10 g/kg BW, respectively, for 10 weeks. The CON and DM groups received an equivalent volume of distilled water via oral gavage concurrently. The dosages of oat β-glucan (0.275, 0.55, and 1.10 g/kg BW) were established based on the effective dose range determined in our previous clinical trial, where daily intake of 50–100 g of naked oats (containing 2.5–5.0 g of β-glucan) significantly improved glycemic profiles in T2DM patients. These doses were translated to the rodent model using body surface area normalization.

### 2.4. The General Conditions, Blood Glucose, Insulin Level and OGTT Analysis

The rats were treated for 10 weeks, during which their general conditions—including food intake, water intake, and body weight—were monitored weekly. Blood samples were collected from the tail tip, and fasting blood glucose levels were measured. Insulin levels were detected after 10 weeks of intervention using standard commercial assay kits following the manufacturer’s protocols. An oral glucose tolerance test (OGTT) was conducted in the 10th week of the intervention. After 12 h of fasting, rats were administered a 50% glucose solution intragastrically at a dose of 2 g/kg BW. Blood samples were collected from the tail tip at 0, 0.5, 1, and 2 h, and blood glucose levels were measured using a microglucometer.

### 2.5. Gut Microbiota Analysis

#### 2.5.1. Plate Count and Identification Method

To investigate the effect of oat β-glucan on the gut microbiota, fecal samples were collected upon sacrifice after the 10-week intervention. The samples were cultured to identify specific bacterial populations, including *Escherichia coli*, *Enterococcus*, *Bifidobacterium*, and *Lactobacillus*, using selective media for each genus. *Escherichia coli* was incubated on eosin-methylene blue (EMB) agar at 37 °C for 24 h. Enterococcus was cultured aerobically on bile salt-aesculin-sodium azide agar at 37 °C for 24 h. *Bifidobacterium* was cultured anaerobically on *Bifidobacterium*-selective (BS) medium at 37 °C for 48 h, and *Lactobacillus* was cultured under anaerobic conditions on *Lactobacillus*-selective (LBS) agar at 37 °C for 48 h. Fecal samples (0.1 g) were collected using sterile tweezers and homogenized in 10 mL of sterile normal saline in UV-sterilized Eppendorf tubes to obtain a 10^−2^ dilution. After vortexing, the suspension was serially diluted 10-fold to prepare concentrations from 10^−2^ to 10^−6^ g/mL. From each dilution, 10 µL, 5 µL, and 2 µL were spotted onto pre-marked regions of selective agar plates, with 2–3 technical replicates per dilution. *Escherichia coli* and *Enterococcus* plates were incubated in an inverted position at 37 °C for 24 h. *Bifidobacterium* and *Lactobacillus* plates were incubated in an inverted position at 37 °C for 48 h under anaerobic conditions generated using a burning candle and an anaerobic bag within a sealed chamber. To avoid colony spreading, plates were first incubated in an upright position for 2 h before inversion. After incubation, plates containing 30–300 colonies were selected for enumeration. Colony-forming units (CFU) per gram of feces were calculated using the following formulas based on plating volume:

CFU/g = average colony number × dilution factor × 100 (for 10 µL plating)

CFU/g = average colony number × dilution factor × 500 (for 2 µL plating)

The final results were log-transformed and expressed as log CFU/g for clarity and comparability. Bacterial identity was confirmed based on colony morphology and microscopic examination via Gram staining. Briefly, colonies were smeared onto glass slides with a drop of sterile saline, air-dried, and heat-fixed. Staining was performed using crystal violet (1 min), iodine solution (1 min), 95% ethanol decolorization (30–60 s), and safranin counterstain (1 min). Slides were examined under oil immersion. Gram-positive bacteria appeared purple; Gram-negative bacteria appeared red.

#### 2.5.2. 16S rRNA Gene Analysis

To remove insoluble impurities and PCR inhibitors, 1 g (wet weight) of cecal content was suspended in 30 mL of 0.1 M sterile sodium phosphate buffer (SPB, pH 7.0). The suspension was vortexed thoroughly for 15 min and centrifuged at 200× *g* for 5 min. The supernatant was collected, and this washing process was repeated three times. The final pooled supernatant was centrifuged at 9000× *g* for 5 min to collect the microbial pellet. Total genomic DNA was extracted using the E.Z.N.A. Soil DNA Kit (Omega Bio-tek, Norcross, GA, USA). According to the protocol, 500 mg of glass beads were used for mechanical lysis. Proteins and humic acids were removed using Buffer P2 and HTR Reagent. The concentration and purity of the extracted DNA were determined using a NanoDrop 2000 spectrophotometer (Thermo Scientific, Waltham, MA, USA), ensuring that the ratio was between 1.8 and 2.0. The V3-V4 hypervariable regions of the bacterial 16S rRNA gene were amplified by PCR using specific primers containing unique barcodes. PCR reactions were performed in triplicate in a 20 μL mixture containing 10 ng of template DNA. To ensure library quality, the number of amplification cycles was kept to a minimum and consistent across samples. The resulting PCR products were separated by 2% agarose gel electrophoresis, purified using a DNA gel recovery kit, and quantified using the QuantiFluor™-ST (Promega, Madison, WI, USA). Purified amplicons were used to construct “Y”-shaped adapter libraries, purified with magnetic beads, and paired-end (PE) sequenced (2 × 300 bp) on an Illumina MiSeq platform (Illumina, San Diego, CA, USA). The remaining high-quality sequences were clustered into operational taxonomic units (OTUs) at a similarity threshold of 97% using UPARSE (v7.0.1090). Taxonomic assignments for each OTU were conducted with the RDP Classifier (v2.7), utilizing the Greengenes database (version 13.8) at a confidence threshold of 0.8. OTU tables and relative abundance tables were generated to quantify the community structure. Alpha- and beta- diversity analyses were conducted based on the OTU data. Alpha-diversity indices, including observed species, Chao1, Shannon, and Simpson indices, were calculated, and rarefaction curves were generated to assess sampling sufficiency. Statistical comparisons among groups were performed using ANOVA. Beta-diversity was evaluated using UniFrac distances (weighted and unweighted), and differences in community structure among groups were visualized via principal coordinate analysis (PCoA). Statistical significance of clustering patterns was tested using PERMANOVA. Linear discriminant analysis Effect Size (LEfSe) was used to identify microbial biomarkers. Spearman correlation analysis was further performed to evaluate the associations between differential gut microbial families identified by LEfSe and inflammatory factors (IL-6, and TNF-α). Correlation coefficients were calculated in R (v4.2.0), and the results were visualized as a heatmap. Statistical significance was indicated as *p* < 0.05 and *p* < 0.01. All statistical analyses and visualizations were performed using R software (v4.2.0), specifically employing the vegan, ggplot2, and phyloseq packages for comprehensive community structure visualizations.

### 2.6. Liver Function Test

Serum concentrations of alanine aminotransferase (ALT), total bilirubin (TBIL), total bile acid (TBA), and alkaline phosphatase (ALP) were measured using an automatic biochemical analyzer 7020 (Hitachi Ltd., Tokyo, Japan).

### 2.7. Liver Histology

Liver biopsies from the rats were fixed in neutral buffered formalin solution (Surgipath Europe, Peterborough, UK) for over 24 h, embedded in paraffin and then sectioned at 4 μm. The liver sections were stained with hematoxylin and eosin (H&E) for histopathological assessment.

### 2.8. Biochemical Indicator Measurements in the Blood

Serum total cholesterol (TC) and triglyceride (TG) levels were measured using an automatic biochemical analyzer 7020 (Hitachi Ltd., Japan) with commercial assay kits (Fir Golden Bridge Biotechnology Co., Ltd., Beijing, China). Fasting plasma glucose (FPG) was measured by spectrophotometry (UV-762, Shanghai Precision Scientific Instrument Co., Ltd. (Shanghai, China)), while low-density lipoprotein cholesterol (LDL-C) and high-density lipoprotein cholesterol (HDL-C) were determined using the automatic biochemical analyzer 7020 (Hitachi, Japan).

### 2.9. Detection of Inflammatory Indicators in the Serum and Liver Tissue

The concentrations of serum lipopolysaccharide (LPS), interleukin-6 (IL-6), and tumor necrosis factor-α (TNF-α) were measured using ELISA kits (Fir Golden Bridge Biotechnology Co., Ltd.,Beijing, China) according to the manufacturer’s protocol. The levels of TNF-α and IL-6, indicators of inflammatory damage in the liver, were also measured using ELISA kits (Andygene Ltd., Beijing, China).

### 2.10. Statistical Analyses

We used SPSS software version 17 (SPSS Inc., Chicago, IL, USA) for statistical analyses. The values were presented as mean ± standard deviation (SD). Differences among groups were analyzed by one-way analysis of variance (ANOVA), followed by the Tukey–Kramer post hoc test for multiple comparisons (when data variances were homogeneous) or the Dunnett’s T3 post hoc test if variances were unequal. Statistical significance was defined as *p* < 0.05.

## 3. Results

### 3.1. Effect of Oat β-Glucano on Body Weight, Urine Volume and Food Intake

**Figure 1:**
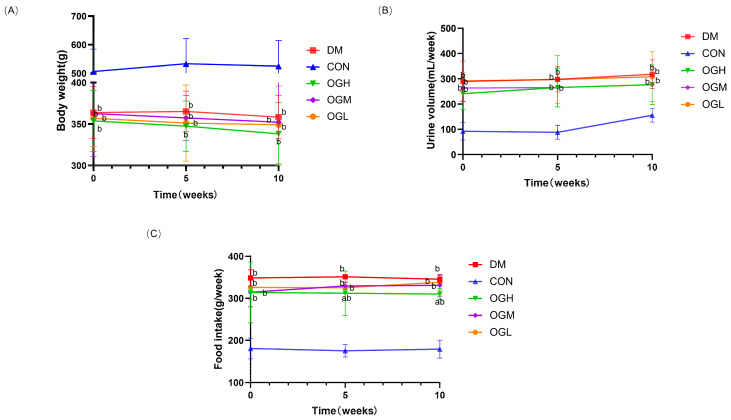
**Effect of oat β-glucan on body weight, urine volume and food intake.** (**A**) Effect of oat β-glucan on body weight. (**B**) Effect of oat β-glucan on urine volume. (**C**) Effect of oat β-glucan on food intake DM: diabetic model; CON: normal control; OGH: Oat β-Glucan High-Dose; OGM: Oat β-Glucan Medium-Dose; OGL: Oat β-Glucan Low-Dose. *n* = 12 ^a^ indicates a statistically significant difference compared to the diabetic model group (*p* < 0.05). ^b^ indicates a statistically significant difference compared to the normal control group (*p* < 0.05).

At weeks 0, 5, and 10 of the intervention, the body weight of rats in the DM group was significantly lower than that in the CON group (*p* < 0.05), aligning with the characteristic symptom of diabetes-induced weight loss. However, no statistically significant differences in body weight were observed between the oat β-glucan intervention group and the DM group, suggesting that oat β-glucan intervention did not alter the body weight of T2DM rats (Figure 1A). Regarding urine volume, the DM group exhibited a significant increase in urine output compared to the CON group (*p* < 0.05). However, there was no statistically significant difference in urine volume among the different oat β-glucan intervention groups when compared to the DM group (Figure 1B). At week 0, the DM group demonstrated a significantly higher food intake compared to the CON group (*p* < 0.05), while no significant differences were found between the oat β-glucan intervention groups and the DM group. However, by weeks 5 and 10, the DM group showed a significantly higher food intake compared to the CON group (*p* < 0.05), and the OGH group exhibited a significantly lower food intake than the DM group (*p* < 0.05) (Figure 1C).

### 3.2. Effect of Oat β-Glucan on Fasting Blood Glucose and Glucose Tolerance

**Figure 2:**
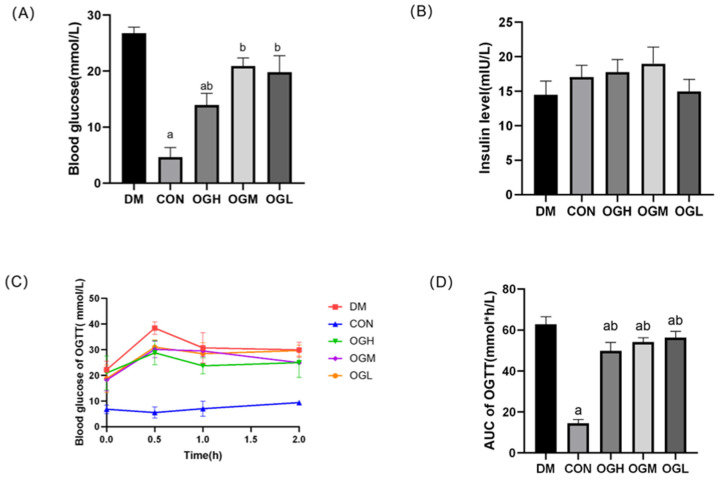
**Effect of oat β-glucan on blood glucose homeostasis in diabetic mice.** (**A**) Fasting blood glucose levels. (**B**) Serum insulin levels. (**C**) Blood glucose profiles during the oral glucose tolerance test (OGTT). (**D**) The area under the curve (AUC) of OGTT. DM: diabetic model; CON: normal control; OGH: Oat β-Glucan High-Dose; OGM: Oat β-Glucan Medium-Dose; OGL: Oat β-Glucan Low-Dose; *n* = 8. ^a^ indicates a statistically significant difference compared to the diabetic model group (*p* < 0.05). ^b^ indicates a statistically significant difference compared to the normal control group (*p* < 0.05).

To evaluate the effect of oat β-glucan on blood glucose levels, we measured fasting blood glucose in the rats after 10 weeks of intervention. As shown in Figure 2A, compared with the DM group, the fasting blood glucose in the OGH group was significantly reduced. This result indicates that oat β-glucan can improve blood glucose levels in diabetic rats. Regarding the serum insulin levels (Figure 2B), following the 10-week oat β-glucan intervention, rats in the OGH and OGM groups exhibited a slight upward trend in insulin levels compared to the DM group; however, no statistically significant differences were detected. The OGTT results further demonstrated the modulatory effect of oat β-glucan on glycemic homeostasis in diabetic rats. As shown in Figure 2C, blood glucose levels in the DM group were significantly higher than those in the CON group at all time points after glucose loading (0, 0.5, 1.0, and 2.0 h). Specifically, the DM group exhibited a marked increase in blood glucose at 0.5 h, which remained elevated throughout the test period. In contrast, the CON group maintained stable and significantly lower blood glucose levels. Treatment with oat β-glucan (OGH, OGM, and OGL) significantly reduced blood glucose concentrations compared with the DM group at most time points, with the most pronounced effect observed in the OGH group. These findings indicate that oat β-glucan intervention effectively attenuated glucose intolerance in diabetic rats. Consistent with the changes in blood glucose profiles, the area under the curve (AUC) of the OGTT (Figure 2D) was significantly larger in the DM group than in the CON group (*p* < 0.05). Compared with the DM group, the OGH, OGM, and OGL treatment groups all showed significantly reduced AUC values (*p* < 0.05).

### 3.3. Histological Analysis

**Figure 3:**
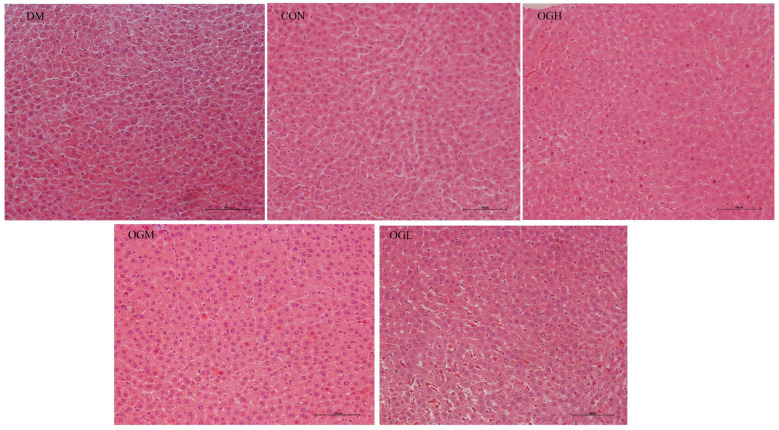
**Histological analysis of liver tissues (H&E staining).** Representative hematoxylin and eosin (H&E)-stained liver sections from different groups. DM: diabetic model; CON: normal control; OGH: Oat β-Glucan High-Dose; OGM: Oat β-Glucan Medium-Dose; OGL: Oat β-Glucan Low-Dose.

Histological examination by H&E staining showed that the liver tissue in the CON group exhibited normal architecture, with well-organized hepatic cords and intact hepatocyte morphology. In contrast, the DM group showed obvious hepatic pathological injury, characterized by disordered hepatic cords, hepatocellular swelling, and cytoplasmic vacuolar degeneration. Oat β-glucan intervention alleviated these pathological changes to varying degrees. The OGL group showed partial improvement, although mild structural disorganization and hepatocellular degeneration were still observed. In comparison, the OGM and OGH groups exhibited more marked restoration of hepatic architecture, with more regularly arranged hepatic cords and reduced hepatocellular swelling and vacuolar changes (Figure 3). These findings indicated that oat β-glucan effectively ameliorated diabetic liver injury.

### 3.4. Effect of Oat β-Glucan on Liver Function and Blood Lipids

**Figure 4:**
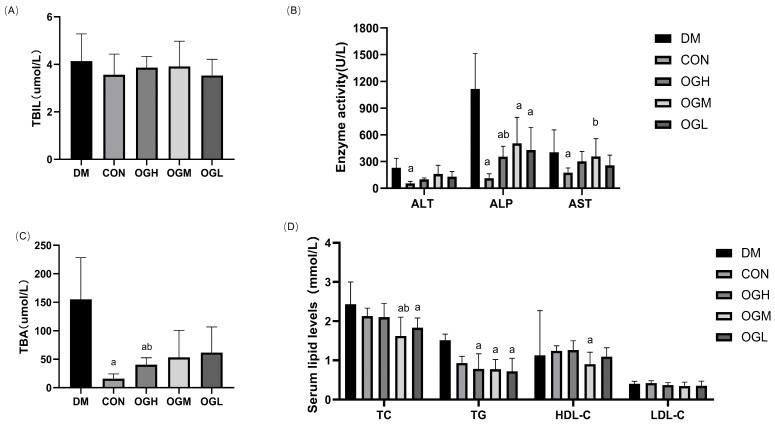
**The effects of oat β-glucan on liver function and serum lipids in diabetic rats**. (**A**) The effects of oat β-glucan on TBIL. (**B**) The effects of oat β-glucan on ALT,ALP,AST. (**C**) The effects of oat β-glucan on TBA. (**D**) The effects of oat β-glucan on TC,TG,HDL-C,LDL-C. DM: diabetic model; CON: normal control; OGH: Oat β-Glucan High-Dose; OGM: Oat β-Glucan Medium-Dose; OGL: Oat β-Glucan Low-Dose. *n* = 8. ^a^ indicates a statistically significant difference compared to the diabetic model group (*p* < 0.05). ^b^ indicates a statistically significant difference compared to the normal control group (*p* < 0.05).

As shown in Figure 4A, there were no significant differences in total bilirubin (TBIL) among all groups. Alanine aminotransferase (ALT) levels were significantly higher in the DM group than in the CON group (*p* < 0.05), while differences among the other groups were not statistically significant. Alkaline phosphatase (ALP) levels in the DM group were significantly elevated compared to all other groups (*p* < 0.05), and oat β-glucan can significantly reduce the ALP level in diabetic rats. Aspartate aminotransferase (AST) levels were significantly higher in the DM and OGM groups than in the CON group (*p* < 0.05), while no significant differences were found among the remaining groups (Figure 4B). Total bile acid (TBA) levels were significantly reduced in both CON group and OGH group compared to the DM group (*p* < 0.05) (Figure 4C). Total cholesterol (TC) levels were significantly lower in the OGL and OGM groups compared to DM group (*p* < 0.05), with a significant reduction also observed in the OGM group compared to the CON group (*p* < 0.05); no significant differences were found among other groups. Triglyceride (TG) levels were significantly decreased in all oat β-glucan dosage groups compared to the DM group (*p* < 0.05). HDL-C concentrations were significantly lower in the OGM group compared to the DM group (*p* < 0.05), with no significant differences among the other groups. There was no significant differences in LDL-C level among all groups (Figure 4D).

### 3.5. The Effects of Oat β-Glucan on Serum LPS, IL-6, and TNF-α Levels in Diabetic Rats

**Figure 5:**
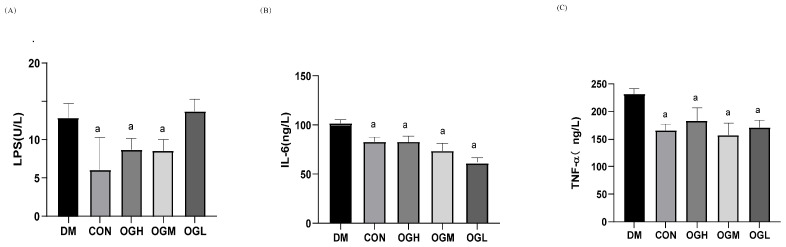
**The effects of oat β-glucan on serum LPS, IL-6, and TNF-α levels in diabetic rats.** (**A**) Serum lipopolysaccharide (LPS) levels; (**B**) Serum interleukin-6 (IL-6) levels; and (**C**) Serum tumor necrosis factor-alpha (TNF-α) levels. *n* = 8, DM: diabetic model; CON: normal control; OGH: Oat β-Glucan High-Dose. OGM: Oat β-Glucan Medium-Dose; OGL: Oat β-Glucan Low-Dose. ^a^ indicates a statistically significant difference compared to the diabetic model group (*p* < 0.05).

Serum LPS levels were significantly decreased in the CON, OGM, and OGH groups compared to the DM group (*p* < 0.05), whereas no significant change was observed in the OGL group. Additionally, serum concentrations of IL-6 and TNF-α were significantly lower in the CON group and all oat β-glucan intervention groups than in the DM group (*p* < 0.05) (Figure 5).

### 3.6. The Effects of Oat β-Glucan on Hepatic Inflammation Markers in Diabetic Rats

**Figure 6:**
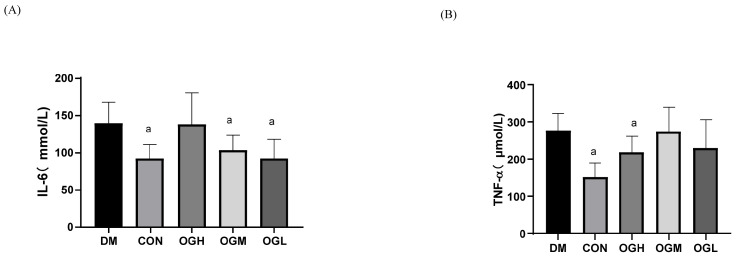
**The effects of oat β-glucan on inflammation markers in diabetic rats**, (**A**) **Hepatic interleukin-6 (IL-6) levels**; (**B**) **Hepatic tumor necrosis factor-alpha (TNF-α) levels.** *n* = 8. DM: diabetic model; CON: normal control; OGH: Oat β-Glucan High-Dose. OGM: Oat β-Glucan Medium-Dose; OGL: Oat β-Glucan Low-Dose. ^a^ indicates a statistically significant difference compared to the diabetic model group (*p* < 0.05).

To further assess the effects of oat β-glucan on hepatic inflammatory cytokines in diabetic rats, we examined the hepatic levels of IL-6 and TNF-α. As shown from Figure 6A, IL-6 levels in the OGM and OGL groups were significantly lower than those in the DM group. Similarly, the TNF-α level in the OGH group was also significantly decreased compared to that in the DM group (Figure 6B). These results suggest that oat β-glucan can alleviate hepatic inflammation in diabetic rats.

### 3.7. Effect of Oat β-Glucan on Lactobacillus, Bifidobacterium, Enterobacterium, and Enterococcus in Fecal Flora Plate Culture

**Figure 7:**
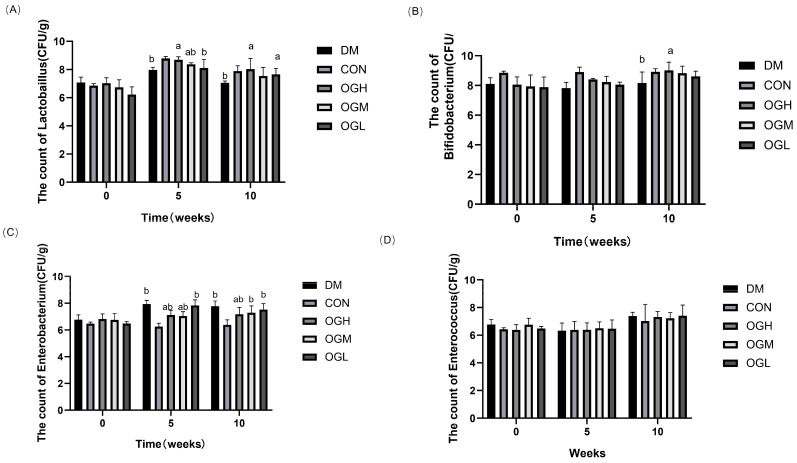
**Effect of oat β-glucan on *Lactobacillus*, *Bifidobacterium*, *Enterobacterium*, and *Enterococcus* in fecal flora plate culture.** (**A**) Effect of oat β-glucan on *Lactobacillus*. (**B**) Effect of oat β-glucan on *Bifidobacterium*. (**C**) Effect of oat β-glucan on *Enterobacterium*. (**D**) Effect of oat β-glucan on *Enterococcus.* DM: diabetic model; CON: normal control; OGH: Oat β-Glucan High-Dose; OGM: Oat β-Glucan Medium-Dose; OGL: Oat β-Glucan Low-Dose. *n* = 12 ^a^ indicates a statistically significant difference compared to the diabetic model group (*p* < 0.05). ^b^ indicates a statistically significant difference compared to the normal control group (*p* < 0.05).

As was shown in Figure 7, at week 0 of the intervention, no statistically significant differences in the gut microbiota were observed among the groups. By week 5, compared to the DM group, the OGH and OGM groups showed a significant increase in the abundance of *Lactobacillus* (*p* < 0.05) and a significant decrease in that of *Enterobacterium* (*p* < 0.05). Subsequently, by week 10, the OGH and OGL groups exhibited a significant increase in *Lactobacillus* (*p* < 0.05) compared to the DM group, while the OGH group also showed a significant increase in *Bifidobacterium* abundance (*p* < 0.05) and a significant decrease in *Enterobacterium* abundance (*p* < 0.05). Throughout the entire experiment, there were no significant changes in *Enterococcus* populations.

### 3.8. The Effects of Oat β-Glucan on Gut Microbiota Composition

**Figure 8:**
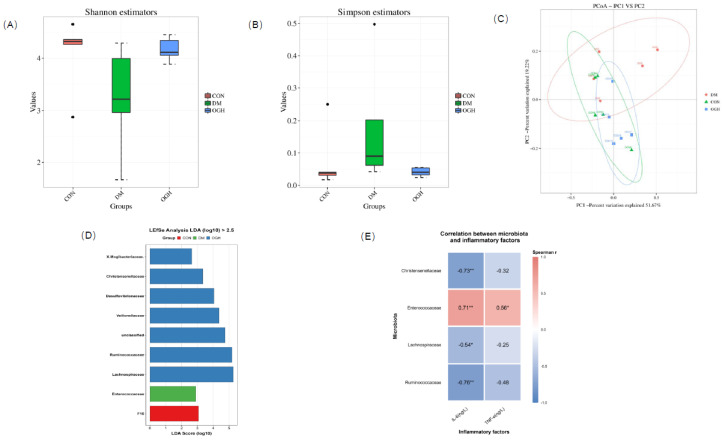
**The effects of oat β-glucan on the gut microbiota.** (**A**) Shannon diversity index for CON, DM and OGH groups. (**B**) Simpson diversity index for CON, DM and OGH groups. (**C**) Principal coordinate analysis (PCoA) of β-diversity (PC1, 51.67%; PC2, 19.22%) showing clustering of CON and OGH apart from DM. (**D**) LEfSe LDA score barplot (LDA > 2.5) identifying discriminative families: F16 for CON; *Enterococcaceae* for DM; and *Lachnospiraceae*, *Ruminococcaceae*, *Veillonellaceae*, *Desulfovibrionaceae*, *Christensenellaceae* and *Mogibacteriaceae* for OGH. (**E**) Spearman correlation heatmap between differential gut microbiota and inflammatory factors. * *p* < 0.05, ** *p* < 0.01 indicate statistically significant correlations between specific microbiota and inflammatory factors. DM: diabetic model; CON: normal control; OGH: Oat β-Glucan High-Dose.

Alpha-diversity analysis, represented by the Shannon and Simpson indices (Figure 8A,B), revealed significant differences in microbial community structure among the groups. Compared with the CON group, the DM group exhibited a marked loss of richness and evenness, with a significantly lower Shannon index and a higher Simpson index (both *p* < 0.05). Following high-dose oat β-glucan treatment, these indices rebounded to levels indistinguishable from the CON group (*p* > 0.05), indicating the restoration of community complexity. These results were further supported by the principal coordinate analysis (PCoA) of β-diversity (Figure 8C). The PCoA plot (PC1 = 51.67% PC = 19.22%) showed that the OGH group clustered closely with the healthy CON group, while both remained distinct from the diabetic model. The close proximity of the OGH and CON clusters in the PCoA plot underscores the potent efficacy of oat β-glucan in restoring the overall gut microbiota profile of diabetic rats to a state that closely resembles healthy controls.

Figure 8D presents the LEfSe-derived LDA score barplot, which identifies the family-level taxa most strongly associated with each experimental group. In the CON group, F16 was the most representative biomarker, indicating its enrichment in healthy controls. In contrast, the DM group was characterized by an increased abundance of *Enterococcaceae*, suggesting a diabetes-associated microbial signature. Notably, OGH treatment selectively enriched several commensal families associated with gut health, including *Lachnospiraceae*, *Ruminococcaceae*, *Veillonellaceae*, *Desulfovibrionaceae*, *Christensenellaceae*, and *Mogibacteriaceae*, all of which exhibited the highest positive LDA scores in the OGH group. The magnitude and distribution of these LDA scores indicate that oat β-glucan not only counteracted the diabetes-associated enrichment of *Enterococcaceae* but also promoted the expansion of butyrate- and propionate-producing bacteria. Collectively, these results demonstrate that OGH reprogrammed the diabetic gut microbiota toward a healthier, Bacillota-enriched community structure.

To further explore the functional relevance of these microbial shifts in the context of disease progression, a Spearman correlation analysis was performed (Figure 8E). We focused on key representative taxa that have been previously implicated in the pathogenesis of diabetes or hepatic injury. Among these, *Enterococcaceae* showed significant positive correlations with IL-6 (r = 0.71, *p* < 0.01) and TNF-α (r = 0.56, *p* < 0.05), suggesting a potential association with a pro-inflammatory state. In contrast, *Christensenellaceae*, *Lachnospiraceae*, and *Ruminococcaceae* were negatively correlated with inflammatory markers, particularly IL-6, with *Christensenellaceae* (r = −0.73, *p* < 0.01), *Lachnospiraceae* (r = −0.54, *p* < 0.05), and *Ruminococcaceae* (r = −0.76, *p* < 0.01) showing significant inverse associations. These findings indicate that oat β-glucan-associated microbial alterations were closely linked to the inflammatory status of diabetic rats, and that the enrichment of beneficial families such as *Lachnospiraceae* and *Ruminococcaceae* may contribute to the anti-inflammatory effects of OGH.

Taken together, these results demonstrate that oat β-glucan markedly reshaped the gut microbiota in diabetic rats, restoring both overall community diversity and taxonomic composition toward a profile more similar to that of healthy controls. In particular, OGH promoted the enrichment of several beneficial microbial families, including *Lachnospiraceae* and *Ruminococcaceae*, while suppressing diabetes-associated microbial signatures. Moreover, the significant correlations between these differential taxa and inflammatory factors suggest that the gut microbiota may be an important mediator through which oat β-glucan alleviates systemic inflammation and improves metabolic disturbances in diabetic rats.

## 4. Discussion

Previous studies have shown that oats can effectively reduce blood glucose levels in humans and experimental models [27]. Consistent with these findings, our study demonstrated that oat β-glucan intervention effectively lowered fasting blood glucose and improved glucose tolerance in diabetic rats (Figure 2), confirming its hypoglycemic potential. Regarding general diabetic manifestations, the DM group exhibited typical symptoms of diabetes, including marked weight loss, polyuria, and hyperphagia. Although oat β-glucan did not significantly reverse weight loss or increased urine volume, OGH intervention significantly suppressed excessive food intake at weeks 5 and 10. These findings suggest that oat β-glucan may not fully restore overall metabolic homeostasis but can improve certain metabolic disturbances associated with diabetes, particularly hyperglycemia and abnormal feeding behavior.

The therapeutic efficacy of oat β-glucan was further evidenced by the restoration of hepatic structural and functional integrity. Histological examination (Figure 3) revealed that DM rats suffered hepatic injury, including hepatocellular swelling, and cytoplasmic vacuolar degeneration. OGH treatment markedly alleviated these pathological alterations. These morphological improvements were strongly supported by the biochemical markers in Figure 4. The DM group showed significant elevations in ALT, AST, ALP, and TBA, reflecting impaired liver function. Crucially, OGH intervention significantly reduced TBA and ALP levels compared to the DM group. Furthermore, oat β-glucan demonstrated a clear lipid-lowering effect, as evidenced by the significant reduction in TG across all dosage groups and TC in the OGL and OGM groups. These findings establish that oat β-glucan may act as a multi-target agent that mitigates both diabetic hepatic injury and dyslipidemia.

The gut microbiota is a critical mediator of host metabolic health [28]. Our study employed both plate culture and 16S rRNA sequencing to capture a comprehensive picture of microbial shifts. Plate cultures (Figure 7) showed that oat β-glucan significantly increased the abundance of beneficial *Lactobacillus* and *Bifidobacterium* while inhibiting the proliferation of potentially pathogenic *Enterobacterium*. Advanced sequencing results further revealed that OGH intervention effectively reversed the loss of microbial alpha-diversity (Shannon and Simpson indices, Figure 8A,B) and reshaped the community structure to resemble that of healthy CON rats (PCoA, Figure 8C). Notably, LEfSe analysis (Figure 8D) identified that OGH selectively enriched several commensal families, including *Lachnospiraceae*, *Ruminococcaceae*, and *Christensenellaceae.* These taxa are well-documented producers of short-chain fatty acids (SCFAs), such as butyrate and propionate, which are essential for maintaining intestinal barrier integrity and suppressing hepatic inflammation via the gut–liver axis. While the enrichment of these SCFA-producing taxa correlates strongly with the observed reductions in systemic inflammation, a limitation of the current study is the lack of direct SCFA quantification. The direct metabolic “bridge” between the microbiota and the liver remains to be fully elucidated through targeted metabolomics.

Chronic low-grade inflammation, often triggered by “metabolic endotoxemia,” is a primary driver of diabetic liver injury [29,30]. Our results support this mechanism: the DM group exhibited elevated serum LPS levels, likely due to the increased proportion of Gram-negative bacteria (such as *Enterobacterium*). OGH intervention significantly lowered serum LPS and systemic inflammatory markers, including IL-6 and TNF-α (Figure 5). Moreover, Spearman correlation analysis (Figure 8E) revealed a significant positive correlation between the diabetes-associated *Enterococcaceae* and pro-inflammatory cytokines (IL-6, TNF-α), while beneficial families like *Ruminococcaceae*, *Lachnospiraceae*, *Christensenellaceae* showed strong inverse associations. Consistent with previous studies, Enterococcaceae has been linked to both diabetes and chronic liver diseases, including hepatitis C virus-related chronic liver disease [31,32]. Previous studies have shown that the abundance of *Ruminococcaceae* and *Lachnospiraceae* is negatively correlated with hepatic LPS and IL-6 levels, but positively correlated with butyrate concentration, suggesting that it may suppress hepatic inflammation through the production of short-chain fatty acids, such as butyrate [33]. *Christensenellaceae* is thought to indirectly participate in the mitigation of liver injury through the regulation of LPS signaling, as well as the improvement of metabolic disturbances and oxidative stress [34]. This confirms that oat β-glucan alleviates liver inflammation by suppressing endotoxin-producing bacteria and promoting anti-inflammatory commensals. Ultimately, this microbial shift translates into reduced hepatic inflammation, as evidenced by the decreased levels of IL-6 and TNF-α in the liver (Figure 6). It is worth noting that the therapeutic mechanism of oat β-glucan should not be viewed solely through the lens of gut microbiota correlations; its macromolecular physical properties play an equally foundational role. As a soluble viscous fiber, oat β-glucan forms a gel-like matrix in the gastrointestinal tract, which physically delays gastric emptying and restricts the intestinal absorption of excessive carbohydrates and lipids. This physical barrier directly blunts metabolic nutrient influx into the portal vein, thereby alleviating hepatic lipid accumulation. Moreover, this viscosity-driven restriction of nutrient absorption in the upper digestive tract concurrently alters the substrate availability in the distal gut, which subsequently shapes the microenvironment for gut microbial fermentation. Therefore, the physical viscosity barrier and the biological gut–liver axis may act in tandem to protect against comprehensive diabetic liver injury.

The dosages of oat β-glucan implemented in this study were directly translated from our group’s prior clinical trial, where daily consumption of 50–100 g of naked oats (providing 2.5–5.0 g of β-glucan) successfully improved glycemic profiles in diabetic patients. Based on standard body surface area conversion, our low (0.275 g/kg) and medium (0.55 g/kg) doses for rats are closely approximate to this clinical range. This study has several limitations. First, although the rat model of a high-fat diet combined with STZ-induced diabetes is suitable for studying diabetes-related metabolic disorders, liver injury, inflammation, and gut microbiota dysbiosis, it does not fully reflect the complexity of human type 2 diabetes. Species differences in metabolism, immune responses, and the gut microbiota may limit the translational relevance of the findings. Second, only male rats were included, which may introduce potential bias and limit the generalizability of the results. Third, although significant associations were observed between microbial changes and inflammatory markers, key functional metabolites such as short-chain fatty acids were not directly measured. Therefore, further studies with direct metabolite measurements and human validation are warranted. Finally, a limitation of this study is that oat β-glucan was administered via intragastric gavage rather than normal oral ingestion. Although gavage was necessary to ensure precise and uniform dosage control, it bypasses the oral phase of digestion and may induce mild stress in rats. Future studies using voluntary dietary incorporation are warranted to better replicate natural human nutrition.

## 5. Conclusions

In conclusion, oat β-glucan effectively alleviated diabetic liver injury and improved metabolic disturbances, potentially through modulation of the gut–liver axis. These beneficial effects were associated with gut microbiota remodeling, characterized by a reduced abundance of *Enterococcaceae* and an increased abundance of beneficial taxa such as *Ruminococcaceae*, *Lachnospiraceae*, and *Christensenellaceae.* Such microbial changes may contribute to the attenuation of metabolic endotoxemia and inflammatory responses, as reflected by lower serum LPS levels and decreased systemic and hepatic pro-inflammatory cytokines, including IL-6 and TNF-α. Collectively, these findings suggest that oat β-glucan may serve as a promising functional dietary strategy for improving liver health and metabolic homeostasis in diabetes.

## Data Availability

The data presented in this study are available on request from the corresponding author. The data are not publicly available due to privacy constraints.
